# Endovascular Intervention of a Mycotic Pseudoaneurysm of Accessory Left Hepatic Artery Arising from the Left Gastric Artery Presenting Secondary to Clostridium difficile Colitis: A Case Report

**DOI:** 10.7759/cureus.7802

**Published:** 2020-04-23

**Authors:** Thomas J Serena, Elias Antypas, Naveen Malay, Eugene Laveroni

**Affiliations:** 1 General Surgery/Vascular Surgery, Beaumont Health, Livonia, USA; 2 Radiology, Beaumont Health, Farmington Hills, USA; 3 Vascular Surgery, Beaumont Health, Farmington Hills, USA

**Keywords:** mycotic pseudoaneurysm, accessory left hepatic artery, case report, endovascular aneurysm repair

## Abstract

Hepatic artery aneurysms have an estimated incidence of approximately 0.002%, of which one half are pseudoaneurysms (PsAs). These typically occur following trauma or liver transplant and are often asymptomatic. An uncommon pathology, mycotic aneurysms or PsAs are those that result as a consequence of infections. The danger in mycotic processes stems from their complications of systemic seeding of infection, rupture, and possible exsanguination. This case reports a mycotic PsA that was found in an accessory left hepatic artery (aLHA) branching from the left gastric artery (LGA). The patient presented with recurrent Clostridium difficile colitis with perforation and was later found to have a left upper quadrant vascular lesion during repeat imaging after failing to progress in their clinical course. After multidisciplinary meetings with vascular surgery and interventional radiology, the patient eventually underwent endovascular coil embolization. This is the first documented case of a mycotic PsA secondary to recurrent C. difficile colitis located in an aLHA branching from the LGA.

## Introduction

A pseudoaneurysm (PsA) refers to a disruption in the arterial wall resulting in blood flow between the tunica media and tunica adventitia. They are referred to as PsAs as they do not contain all layers of the arterial wall, excluding the intima [1-2]. Aneurysms of all types receive the descriptor “mycotic” when they form secondary to an infection. Mycotic aneurysms are important pathophysiological processes as they can result in systemic sepsis, arterial rupture, and exsanguination. As a rare clinical entity, infected aneurysms occur in descending order of frequency in the aorta, peripheral arteries, cerebral arteries, and last visceral arteries [3]. Hepatic artery pseuodaneurysms often form secondary to trauma or after liver transplant. Arterial aneurysms of the visceral arteries occur with an incidence as low as 0.1% in autopsy series [4]. Less common are hepatic artery aneurysms which have an estimated incidence of approximately 0.002%, of which one half are PsAs [5]. The cornerstone of treatment for these aneurysms is guided by their cause. The treatment for intrahepatic aneurysms involves coil embolization. However, in mycotic PsAs, surgical resection is the routine treatment as the coils may serve as a source of recurrent infection [6]. This case report presents an accessory left hepatic artery (aLHA) mycotic PsA presenting in conjunction with recurrent transverse colitis secondary to *Clostridium difficile* infection with perforation. This case was diagnosed as a result of clinical examination and CT and then managed by endovascular intervention at a community-based residency program. This case is unique as it is not only a deviation from the standard of care for mycotic PsAs, but it is also the first documented case of a mycotic PsA of an aLHA. This case is reported in accordance with SCARE Criteria [7].

## Case presentation

A 71-year-old Caucasian female presented to the ED after unremitting bilateral lower abdominal pain of acute onset. She also noted slowly worsening, diffuse abdominal pain. She describes the pain as sharp in nature and unrelenting. Past medical history is significant for *C. difficile* colitis in a previous hospital admission. Past surgical history was significant for an appendectomy. The patient’s father and brother both have been diagnosed with colorectal carcinoma. She denies any history of alcohol, tobacco, or illicit drug use.

**Figure 1:**
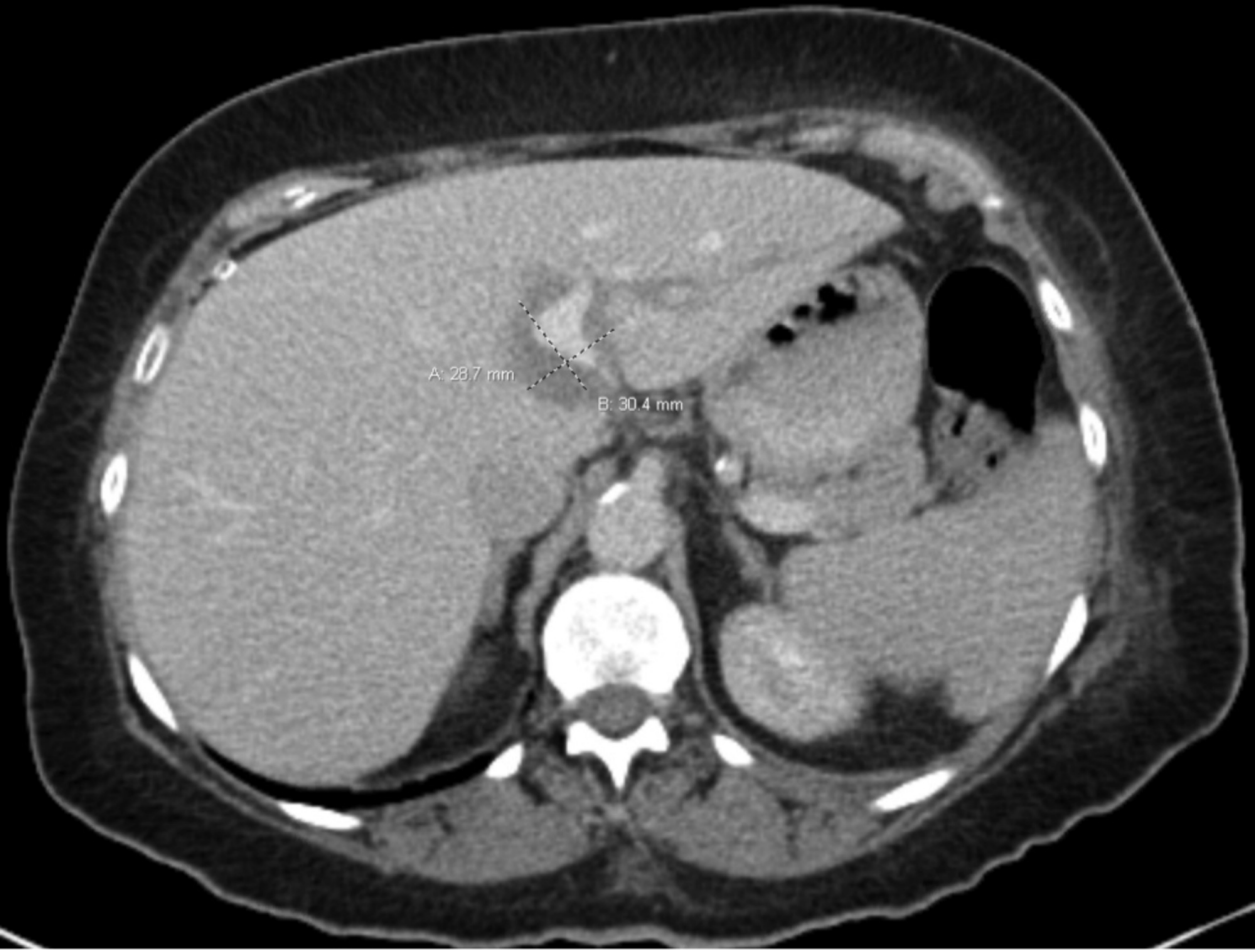
Diagnostic CT. Pre-intervention CT performed after IR drainage of pelvis abscesses revealing a large hypodense lesion in the left lobe of the liver with partial enhancement, as measured on imaging.

**Figure 2:**
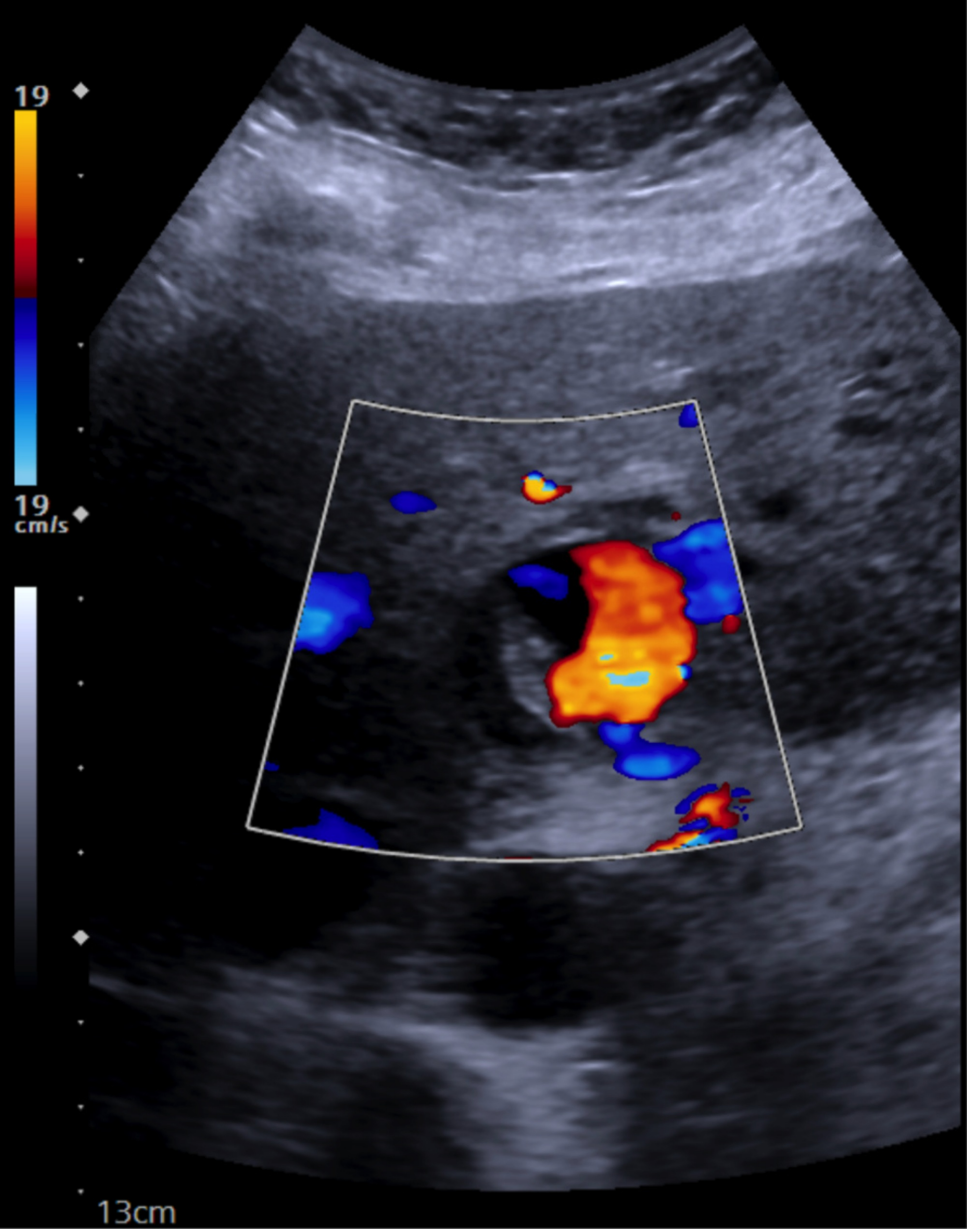
Diagnostic vascular hepatic duplex imaging. Confirmation of pseudoaneurysm (PsA) with demonstration of a cystic structure coursing near an artery with brisk internal color flow and a “to and from” pattern or classic “Ying-Yang” sign.

On initial presentation, all vitals were found to be within normal parameters. The patient’s abdomen was soft, nondistended, but with exquisite tenderness in the bilateral lower quadrants with the addition of diffuse subjective abdominal pain. Laboratory testing was significant for a leukocytosis of 18.6 bil/L. CT was performed revealing two pelvic fluid collections with peripheral enhancement in addition to a mild pneumoperitoneum. Based on physical examination and lack of SIRS criteria, the patient was admitted for recurrent *C. difficile* infection and treated with both broad spectrum antibiotics. A consultation was placed to interventional radiology and the patient underwent CT-guided drainage of multiple pelvic abscesses which were later positive for clostridium species. The patient began to progress, however, then had recurrence of severe abdominal pain coupled with elevating leukocytosis. At this time, repeat CT demonstrated interval development of small pockets of portal venous air in addition to hypodense lesions in the left lobe of the liver with partial enhancement as shown in Figure 1. Due to the rapid development of these lesions, vascular portal duplex was performed revealing thrombus within a hepatic structure with present blood flow, concerning for a partially thrombosed PsA versus arterioportal shunt as shown in Figure 2.

**Figure 3:**
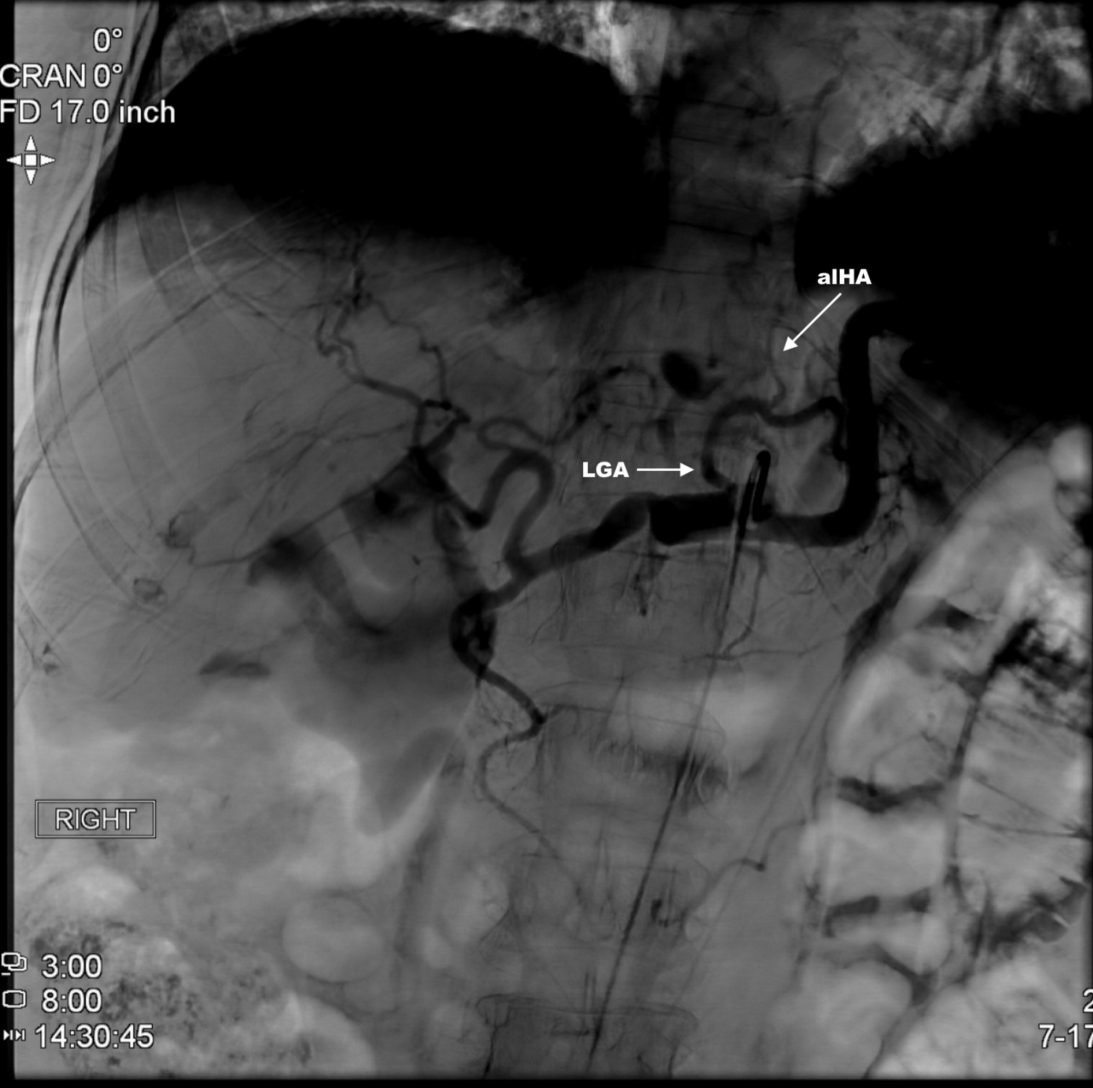
Selective angiography of CT. Selective angiography of celiac trunk (CT) revealing accessory left hepatic artery (aLHA) off left gastric artery (LGA).

**Figure 4:**
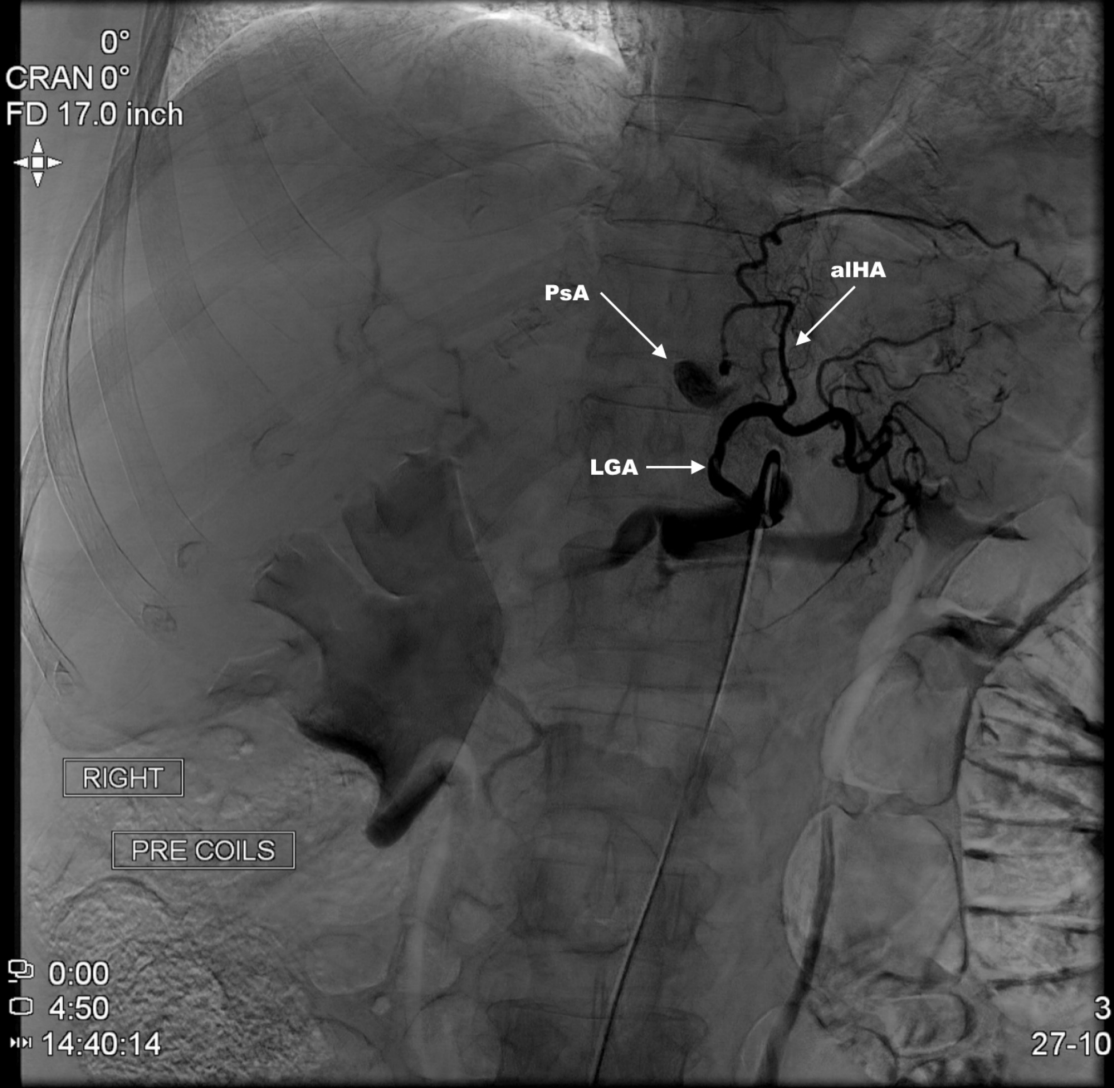
Microcatheter highly selective angiography of LGA. Demonstration of large pseudoaneurysm (PsA) arising off the accessory left hepatic artery (aLHA) from left gastric artery (LGA).

**Figure 5:**
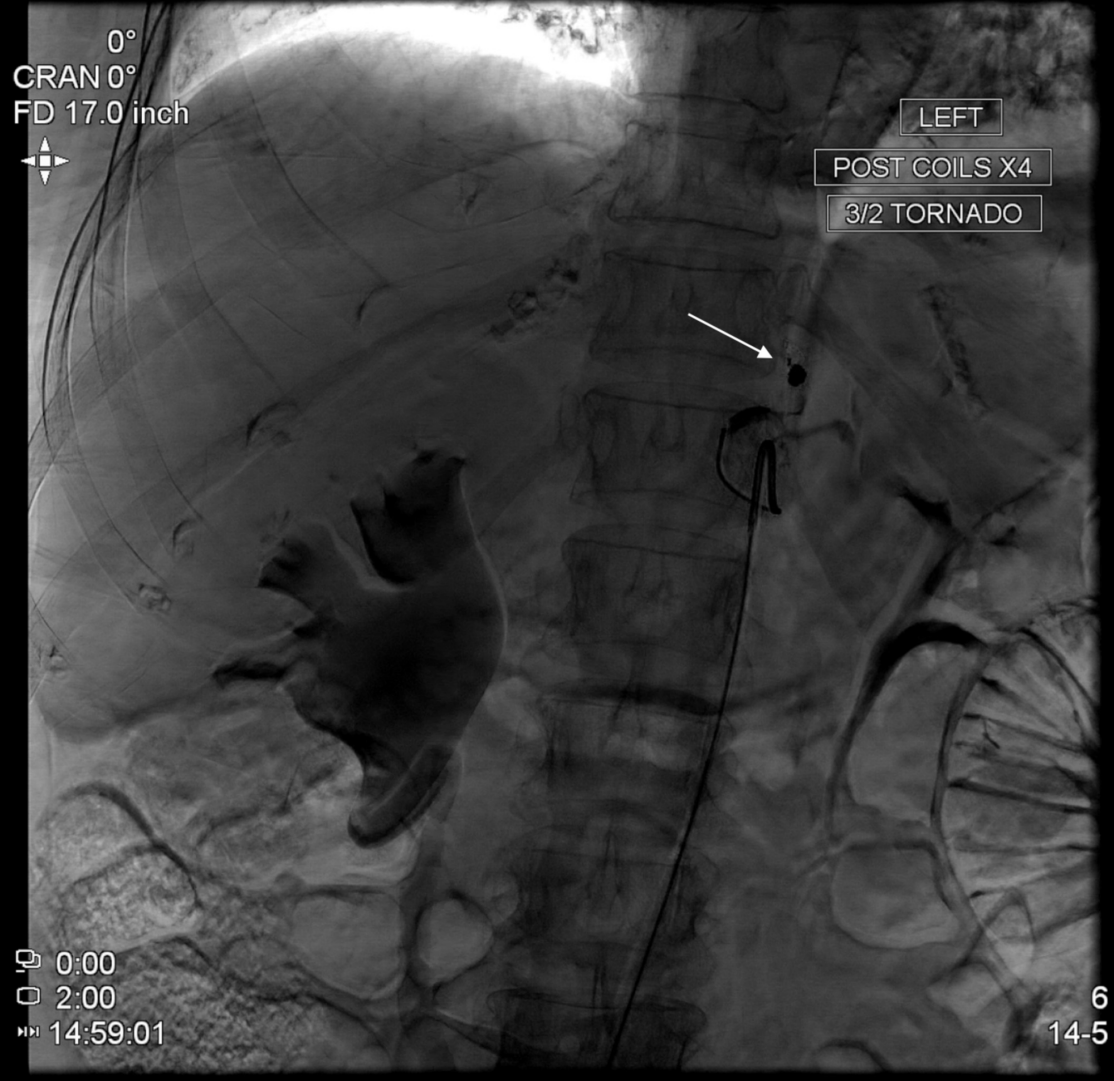
Catheter-directed intervention. Tornado embolization coiling of accessory left hepatic artery (aLHA) off the left gastric artery (LGA), as demonstrated by arrow.

**Figure 6:**
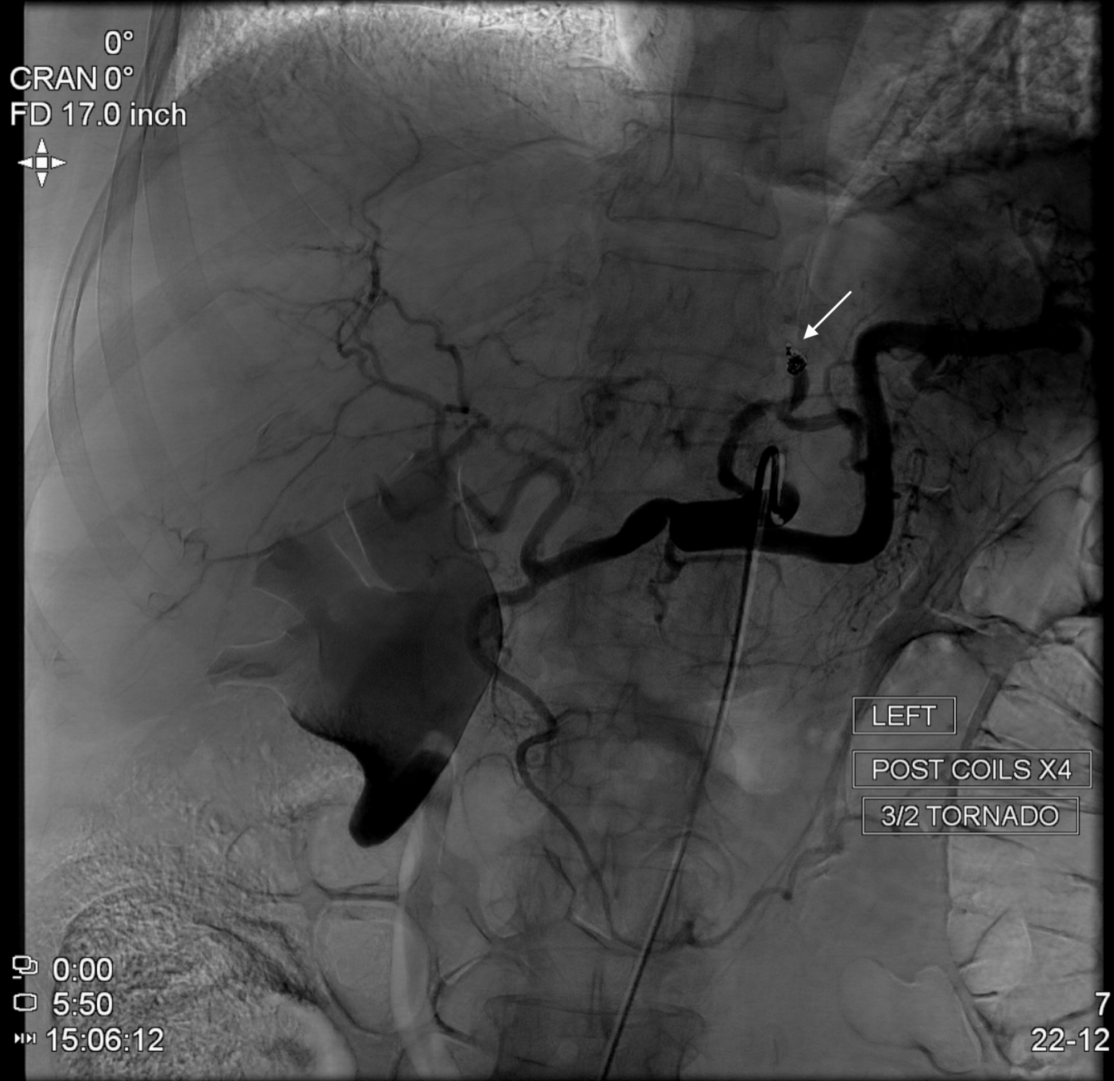
Completion angiography. Catheter-directed completion angiography demonstrating filling defect of feeding vessel to pseudoaneurysm (PsA), as demonstrated by arrow.

Multiple interdisciplinary discussions were conducted between vascular surgery, internal medicine, and the interventional radiology team. Based on the current literature and the location of this PsA, the patient was consented for and underwent endovascular intervention. The interventional team performed selective angiography of the celiac trunk (CT) followed by highly selective targeting of what was found to be an aLHA branch off the LGA as seen in Figure 3. After targeting this vessel, angiography revealed the presence of a PsA as shown in Figure 4. Four tornado coils were deployed followed by loss of opacification of the feeding vessel. The embolization is demonstrated by an arrow in Figure 5. Following the intervention, completion angiography was performed as seen in Figure 6 which reveals a filling defect of the feeding vessel to the PsA. The patient was then transported from the operating suite back to the general medical floor.

 

**Figure 7:**
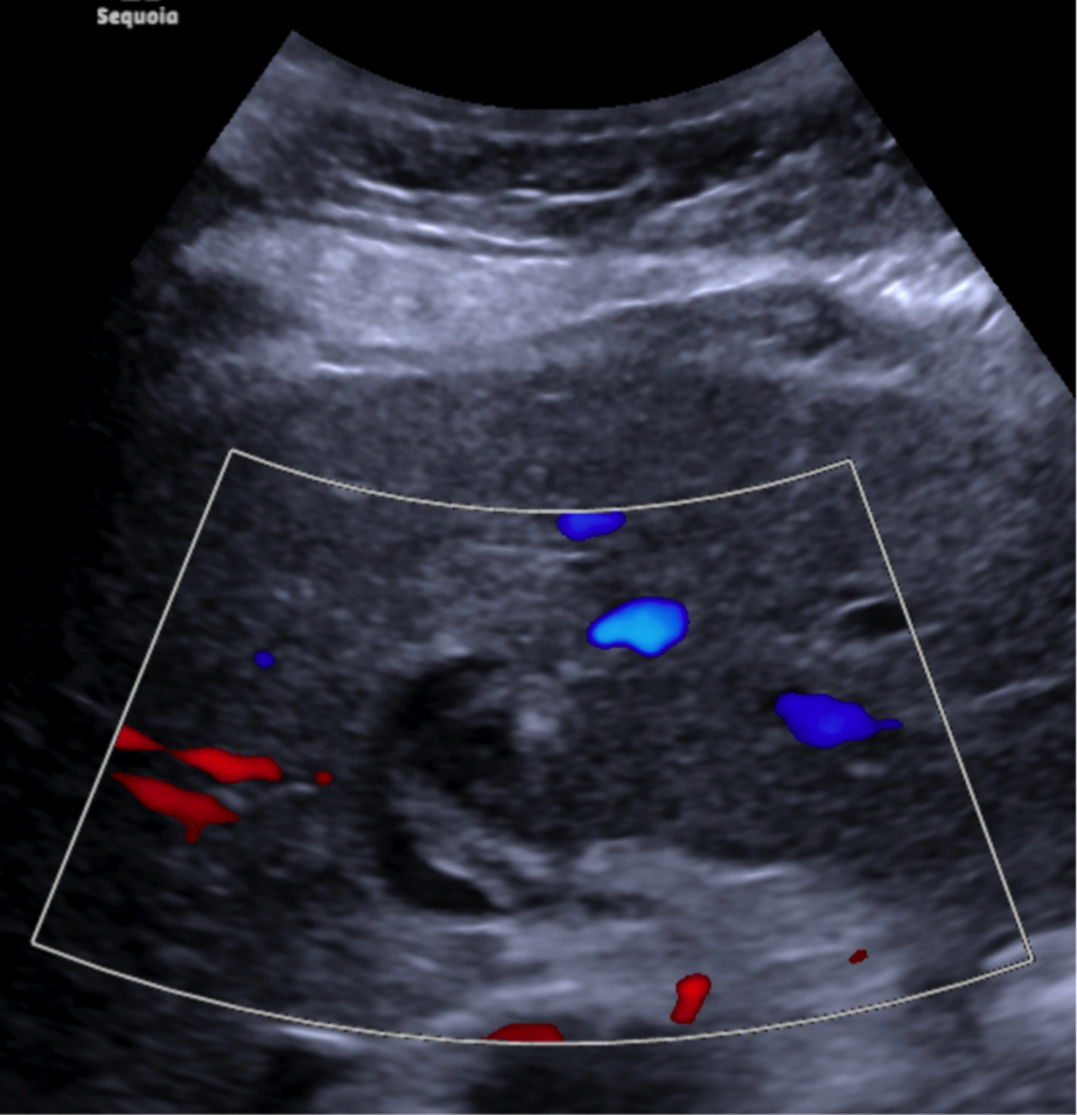
Mesenteric arterial duplex ultrasound. Post-operative imaging demonstrated a pseudoaneurysm (PsA) with heterogeneity to the echogenic material and no discernible blood flow, consistent with successful embolization of the PsA.

The postoperative period was uneventful and the patient remained stable throughout its entirety. On postoperative day one, the patient underwent mesenteric arterial duplex ultrasound that demonstrated a PsA with heterogeneity to the echogenic material and no discernible blood flow, consistent with successful embolization of the PsA as seen in Figure 7. Over the next few days, the patient slowly had resolution of abdominal pain and was discharged from the hospital. The patient has been seen in outpatient follow up and is doing well since surgery and without any complaints.

## Discussion

Pseudoaneurysms are often asymptomatic and found incidentally during investigation of other conditions. Specifically, hepatic artery aneurysms are estimated to have an incidence of approximately 0.002%, most common after transplant or trauma [1]. The less common mycotic aneurysms or PsAs may manifest with local or systemic signs such as abdominal pain or SIRS criteria [6]. Hepatic artery PsAs can be especially fatal due to increased risks of life-threatening hemorrhage. They can be both intrahepatic or extrahepatic. While often found incidentally, imaging with ultrasound can confirm by demonstrating a cystic structure coursing near an artery with brisk internal color flow, and a ‘to and from” pattern or “Ying-Yang” sign. The incidence of hepatic artery aneurysm rupture can be as high at 21%-80%, necessitating intervention [7-9].

There have been multiple studies regarding mycotic aneurysm management, however, the majority of such deal with the aorta, iliac, and femoral arteries. Management differs tremendously when it regards hepatic arteries secondary to difficulty in access for open repair [10]. Treatment options for mycotic aneurysms often include in-situ grafting, resection of aneurysm with remote bypass grafting, or ligation. However, these approaches are better served for locations with ease of access. The treatment of nonmycotic aneurysms and PsA has turned towards endovascular repair for which there has been great success. These options include US-guided percutaneous thrombin injection or endovascular management [8]. The specific approach utilized in this case was chosen second to the intrahepatic nature of the mycotic PsA and poor candidacy for open approach. In addition, endovascular approach to hepatic artery aneurysms is associated with lower morbidity and shorter hospital stays with 86% success rate. Recurrence of hepatic artery aneurysms occurs secondary to recanalization of the artery, which can be managed with repeat embolization or definitive surgical repair [11-12].

**Figure 8:**
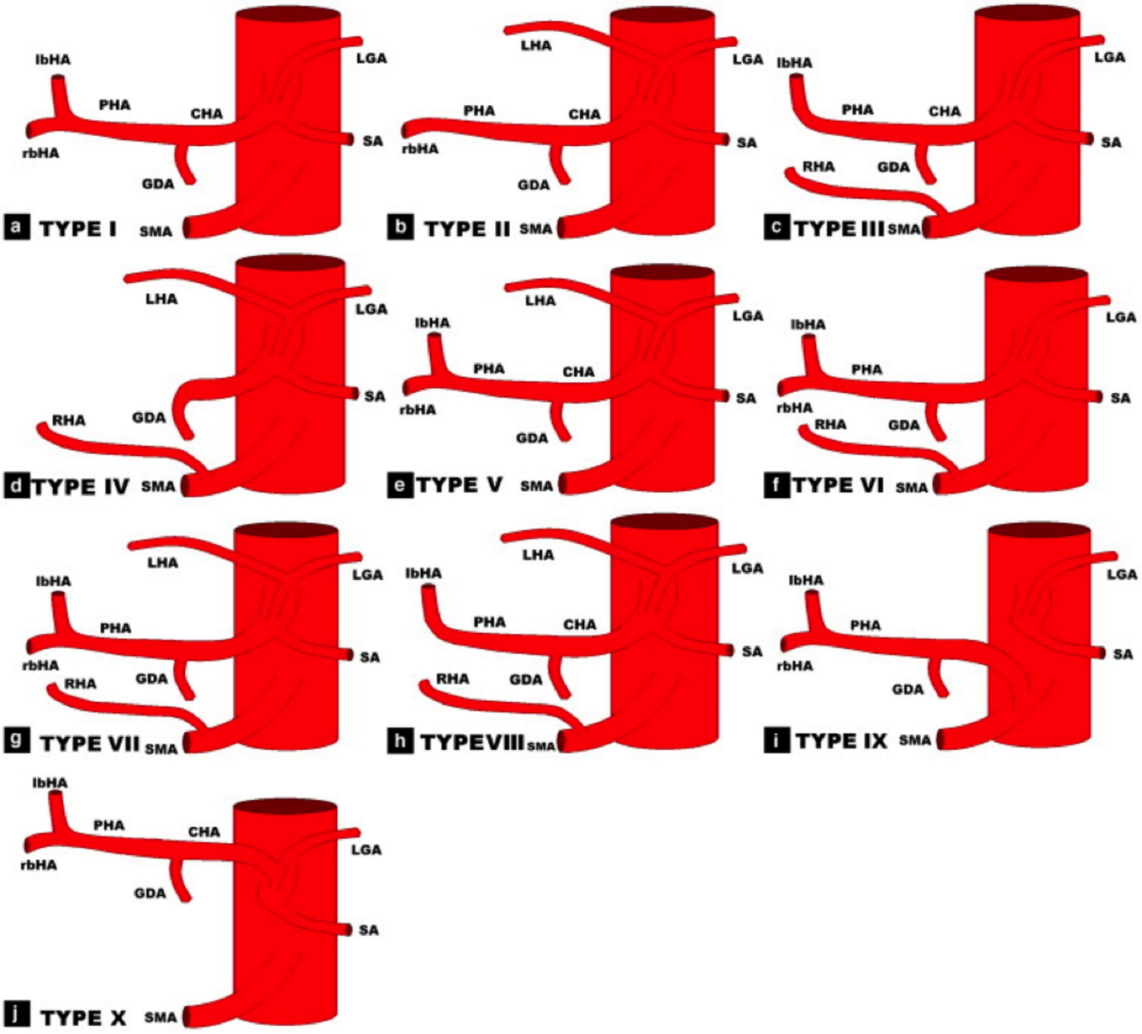
Michels classification system. Classification system for hepatic arterial tree anomalies as presented by Shukla et al. demonstrating anatomy of left gastric artery (LGA), splenic artery (SA), common hepatic artery (CHA), gastroduodenal artery (GDA), superior mesenteric after (SMA), proper hepatic artery (PHA), right hepatic artery (RHA), left hepatic artery (LHA), left branch of hepatic artery (lbHA), and right branch of hepatic artery (rbHA) [13].

**Figure 9:**
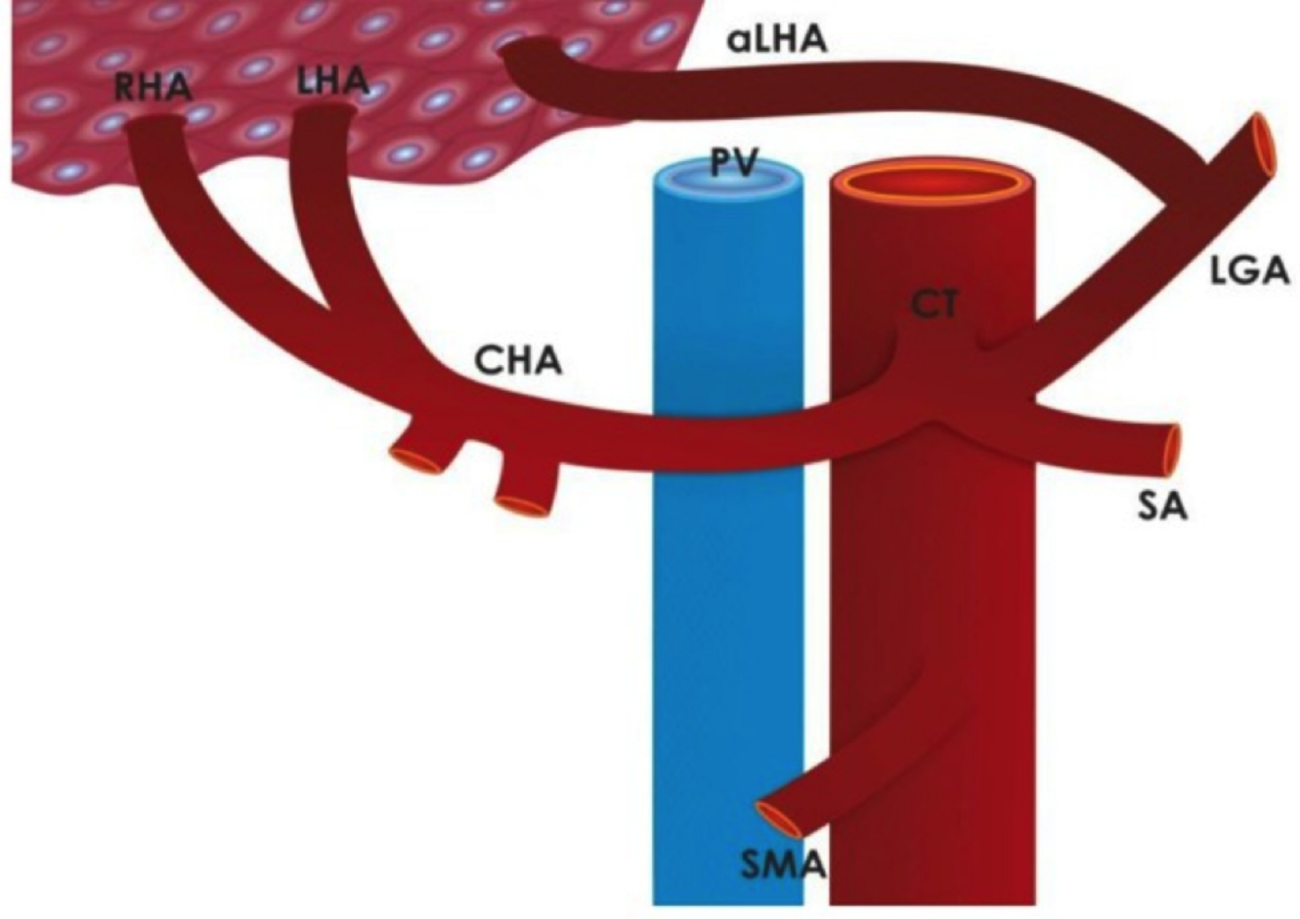
Schematic representation of Type V Michels. Type V an accessory left hepatic artery (aLHA) according to Michels (3.2% of cases). RHA, right hepatic artery; LHA, left hepatic artery; CHA, common hepatic artery; CT, celiac trunk; PV, portal vein; LGA, left gastric artery; SA, splenic artery; SMA, superior mesenteric artery; aLHA, accessory left hepatic artery [15].

In addition to the unconventional nature of the intervention provided, this case is unique as it is the first documented case of an aLHA mycotic PsA. The incidence of hepatic arterial tree anomalies can be as high as 11%-21% of right hepatic arteries (RHAs) and 3.8%-10% of LHAs. However, more rare are aRHAs and aLHAs which occur in 0.8%-8% of cases [13]. Multiple classification systems have been developed for hepatic arterial tree anomalies. The most commonly used is the Michels classification, which has 10 different anomalies as depicted in the Figure 8 [14]. The case presented has a Michels Classification Type V anomaly as further demonstrated in Figure 9, which describes the presence of an LHA originating from the LGA and the left branch of the hepatic artery (lbHA). This occurs in 8% of patients when an anatomical variant is present [15].

Mycotic aneurysms secondary to toxigenic *C. difficile* infections have been written about only in case reports. This infectious process rarely exhibits extraintestinal pathology. The *Journal of Clinical Infectious Disease* estimated the incidence to occur in around 0.17% of all *C. difficile* infections [16]. When manifesting in the abdomen, they occur secondary to a perforation or after some sort of gastrointestinal surgery. One case report presented a patient with a mycotic abdominal aortic aneurysm treated with aorto-bifemoral bypass, as opposed to surgical resection. This patient required four weeks of outpatient antibiotics and was treated with great success [17]. However, currently given the exquisite rarity of this disease process no standard of care for treatment exists.

The highpoint of this case report is how it connects two very rare clinical entities. The first being the presence of a Michels Type V accessory LHA. This couples with a mycotic PsA secondary to transposition from *C. difficile* colitis with perforation. Furthermore, the management of this mycotic PsA was done with an unconventional approach secondary to its poorly accessible deep intrahepatic location. Potential risks of this approach are recurrence and the possibility of further infectious process secondary to the coils being a nidus for infection. If this occurs, the patient will need hepatic resection or complex intrahepatic surgical repair.

## Conclusions

This case report presents an accessory LHA mycotic PsA secondary to recurrent transverse colitis secondary to *C. difficile* infection. This case is further unique as it is not only a deviation from the standard of care for mycotic PsAs, but it is also the first documented case of a mycotic PsA of an aLHA. Given its unfavorable location for open surgical procedure or ultrasound percutaneous thrombin injection, the decision was made to coil this lesion and treat with long-term antibiotics with close outpatient follow up. This case highlights how an interdisciplinary approach serves the best interests of the patient and eventually resulted in a great outcome.
